# Novel ACE Inhibitory Peptides Derived from Simulated Gastrointestinal Digestion in Vitro of Sesame (*Sesamum indicum* L.) Protein and Molecular Docking Study

**DOI:** 10.3390/ijms21031059

**Published:** 2020-02-05

**Authors:** Ruidan Wang, Xin Lu, Qiang Sun, Jinhong Gao, Lin Ma, Jinian Huang

**Affiliations:** Research Center for Agricultural and Sideline Products Processing, Henan Academy of Agricultural Sciences, Zhengzhou 450002, China; wangrd199403@163.com (R.W.); xinlu1981@foxmail.com (X.L.); qiangsunxy@126.com (Q.S.); gaji.good@163.com (J.G.); malin199003@163.com (L.M.)

**Keywords:** sesame protein, ACE inhibitory peptides, simulated gastrointestinal digestion, amino acid sequence, molecular docking

## Abstract

The aim of this study was to isolate and identify angiotensin I-converting enzyme (ACE) inhibitory peptides from sesame protein through simulated gastrointestinal digestion in vitro, and to explore the underlying mechanisms by molecular docking. The sesame protein was enzymatically hydrolyzed by pepsin, trypsin, and α-chymotrypsin. The degree of hydrolysis (DH) and peptide yield increased with the increase of digest time. Moreover, ACE inhibitory activity was enhanced after digestion. The sesame protein digestive solution (SPDS) was purified by ultrafiltration through different molecular weight cut-off (MWCO) membranes and SPDS-VII (< 3 kDa) had the strongest ACE inhibition. SPDS-VII was further purified by NGC Quest™ 10 Plus Chromatography System and finally 11 peptides were identified by Nano UHPLC-ESI-MS/MS (nano ultra-high performance liquid chromatography-electrospray ionization mass spectrometry/mass spectrometry) from peak 4. The peptide GHIITVAR from 11S globulin displayed the strongest ACE inhibitory activity (IC_50_ = 3.60 ± 0.10 μM). Furthermore, the docking analysis revealed that the ACE inhibition of GHIITVAR was mainly attributed to forming very strong hydrogen bonds with the active sites of ACE. These results identify sesame protein as a rich source of ACE inhibitory peptides and further indicate that GHIITVAR has the potential for development of new functional foods.

## 1. Introduction

Hypertension is one of the diseases with the highest mortality in the world, and it is the main pathogenesis factor of coronary heart disease, stroke, and heart and kidney failure [1,2,3]. According to epidemiological studies, more than one billion people suffer from hypertension nowadays [4]. Therefore, the prevention and treatment of hypertension has become a difficult task for the global medical community.

Relevant studies have shown that human blood pressure is regulated by many factors, among which renin-angiotensin system (RAS) and kallikrein kinin system (KKS) are the main ways to control the stability of blood pressure. ACE is a dipeptide carboxyl metalloproteinase which plays a key role in these two systems. In the RAS system, ACE can convert the inactive angiotensin I into angiotensin II, which has the function of constricting vascular smooth muscle, thus, increases the blood pressure. Meanwhile, ACE can inactivate the vasodilator bradykinin in the KKS system, leading to vasoconstriction, which causes an increase in the blood pressure [5]. Currently, many synthetic ACE inhibitors such as captopril, enalapril, alacepril, and lisinopril can effectively reduce high blood pressure. However, these drugs have severe adverse effects on patients such as persistent cough, taste distortion and skin rashes in long term usage [6,7]. Therefore, it is crucial to develop more effective bioactive agents or functional foods as substitutes of synthetic drugs in the prevention and treatment of hypertension. 

Bioactive peptides are small fragments of food protein, mostly composed of 2–20 amino acid residues. They have the characteristics of easy absorption, low sensitivity and good solubility. Meanwhile, they have the function of nutrition and physiological regulation, which have obvious effects on the conditioning and treatment of modern chronic diseases and sub-health state. During processing, enzymatic hydrolysis, fermentation, or gastrointestinal digestion, foods can release bioactive peptides [8,9] which have been reported to exhibit different biological activities including antioxidant [10], antibacterial [11], inhibition activity on dipeptidyl peptidase IV [12], inhibition activity on ACE [13], and so on. So far, lots of ACE inhibitory peptides have been isolated from various food proteins such as milk protein [14], egg protein [15], and marine protein [16]. 

Sesame (*Sesamum indicum* L.) is one of the important oil seed crops worldwide and it is widely used in food, health care, and medical applications because of its high nutritional value [17,18]. Sesame seeds are mainly used to produce sesame oil due to high content of unsaturated fatty acids and lignans. In addition, sesame meal containing almost 50% proteins could be a valuable source of proteins for comprehensive use. Sesame protein has been reported to have ACE inhibitory peptides. Nakano et al. [19] have isolated six ACE inhibitory peptides from sesame protein hydrolyzed by thermolysin. However, there was little information about the ACE inhibitory peptides of sesame protein hydrolysate via simulated gastrointestinal digestion in vitro and molecular docking study. Here, the purpose of this study was to find the changing rules of ACE inhibitory peptides generated from sesame protein during simulated gastrointestinal digestion in vitro and to isolate and identify the sequence of new peptides. Moreover, the binding interaction of the screened ACE inhibitory peptide within the enzymatic active site was further studied through molecular docking simulation. Our results are expected to provide more evidence for the utility of sesame as a functional food for the treatment of hypertension.

## 2. Results

### 2.1. Changes of ACE Inhibitory Activity during Simulated Gastrointestinal Digestion

**Figure 1:**
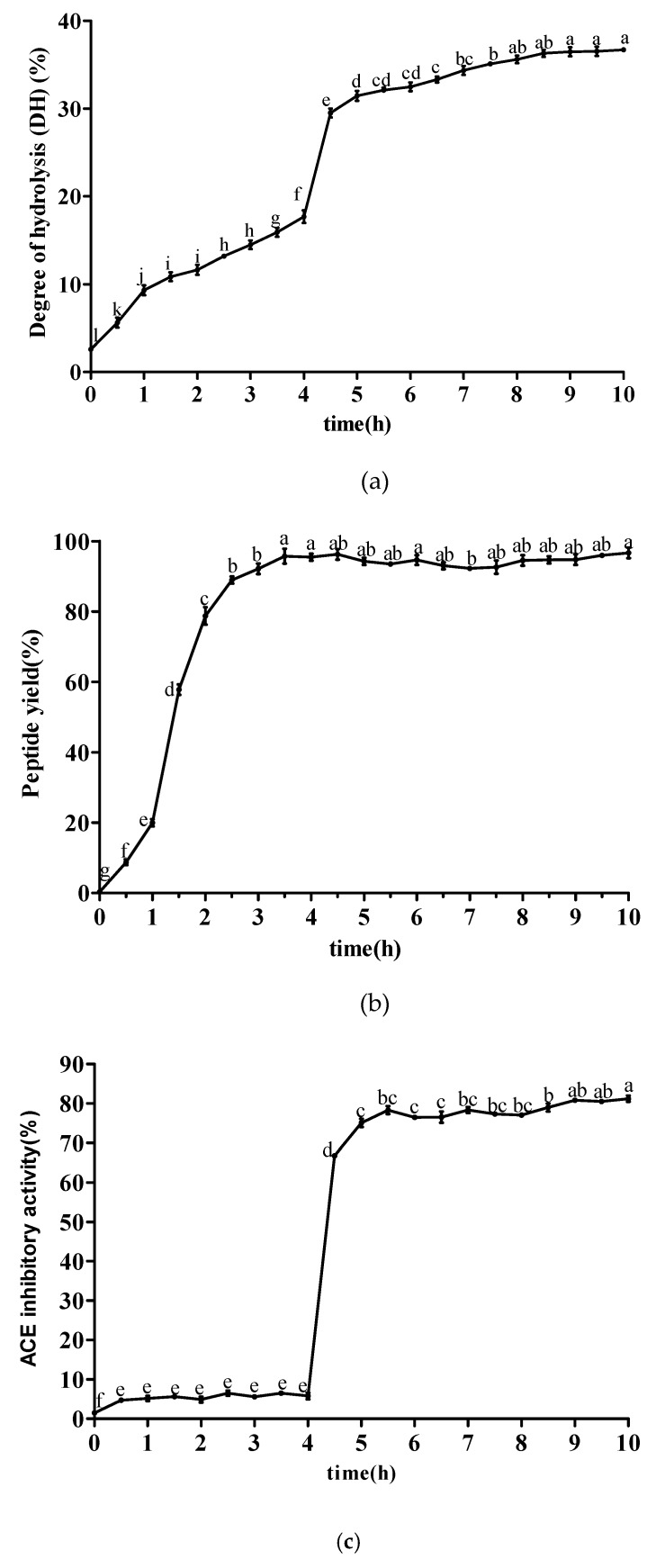
The changing rules of ACE inhibitory peptides at different time points during simulated gastrointestinal digestion. (**a**) Degree of hydrolysis changes at different time points during simulated gastrointestinal digestion. (**b**) The changes of peptide yield at different time points during simulated gastrointestinal digestion. (**c**) The changes of angiotensin I-converting enzyme (ACE) inhibitory activity at different time points during simulated gastrointestinal digestion. Data are expressed as the mean ± standard deviation (*n* = 3) and different letters marked are significantly different by one-way analysis of variation multiple test (*p* < 0.05).

The degree of hydrolysis (DH) represents the extent of protein degradation, which has been widely used in hydrolysis efficiency assessments. As shown in Figure 1a, the DH of sesame protein showed an overall rising trend when simulating gastrointestinal digestion in vitro. In the stage of gastric digestion, sesame protein began to be hydrolyzed, and the DH ranged from 2.59% to 17.69% at 0–4 h. However, the DH increased suddenly when trypsin and α-chymotrypsin was added. In the stage of intestinal digestion, the DH increased slowly and tended to be stable due to the decrease of protein substrates and enzyme cutting sites in the digestive system. The DH eventually reached 36.70%. Figure 1b shows the changes of peptide yield at different time points during simulated gastrointestinal digestion. After adding pepsin, the peptide yield increased significantly ranging 95.46% with the increase of time. After being treated by trypsin and α-chymotrypsin, the peptide yield remained basically stable over a 6 h period. The changing rules of ACE inhibitory activity at different time points during simulated gastrointestinal digestion are shown in Figure 1c. Gastric digestive products of sesame protein exhibited weak ACE inhibitory activity without obvious upward or downward trend at 0–4 h, but intestinal digestive products had strong ACE inhibitory activity at 4–10 h and tended to be stable gradually. The ACE inhibitory activity reached 81.21% at 10 h. It was suggested that pepsin had less ability to hydrolyze sesame protein to produce polypeptide in simulated gastric digestion. There were more ACE inhibitory peptides generated from simulated intestinal digestion, which implied that trypsin and α-chymotrypsin offered the ability to achieve stronger ACE inhibitory peptides. It also would be supposed that the peptide sequences were buried deeper in the original protein structures from which they come or the sequences were embedded in the structured parts in which they were forced to be in the conformations that could not fit the active site of ACE. These results were also similar to that of other reports [20,21,22,23]. 

### 2.2. Separation of ACE Inhibitory Peptide from SPDS by Ultrafiltration

It is well known that molecular weights of peptide fragments are crucial for their biological activities. Ultrafiltration is a membrane separation technique that is of great use in the concentration, purification, fractionation, and clarification of solutes. It has high throughput of product, low operational cost, energy saving potential, and ease of equipment cleaning. In the separation of polypeptides, ultrafiltration can effectively remove insoluble substrates, large molecular weight proteins and peptides, and obtain bioactive peptides with smaller molecular weight [22,24]. The SPDS was subjected to fractionation by ultrafiltration through 100, 50, 30, 10, 5, and 3 kDa MWCO membranes to get seven fractions with >100, 50–100, 30–50, 10–30, 5–10, 3–5, and <3 kDa. The IC_50_ values inhibiting ACE activity were calculated from the results of a series of concentrations. As shown in Table 1, the IC_50_ values of these fractions were 35.143 ± 1.122, 15.066 ± 0.042, 9.146 ± 0.005, 6.108 ± 0.001, 5.106 ± 0.003, 4.583 ± 0.003, 2.720 ± 0.003 μg/mL, respectively. A lower IC_50_ value corresponds to a stronger inhibitory activity. SPDS-VII with molecular weight <3kDa exhibited the strongest ACE inhibitory activity with the lowest IC_50_ value among these fractions, indicating that lower molecular weight peptides or small molecules showed stronger ACE inhibition. This result was in agreement with previous studies reporting that most ACE inhibitory peptides derived from food protein were generally short sequences containing 2–20 amino acids, or the hydrolyzates were generally filtered through a 3 kDa membrane [25,26]. In addition, the high molecular weight fraction could not enter the ACE active site easily. Therefore, it was hard to change the catalytic activity of ACE compared with the low molecular weight fraction [27].

### 2.3. Purification of ACE Inhibitory Peptide by NGC Quest™ 10 Plus Chromatography System

**Figure 2:**
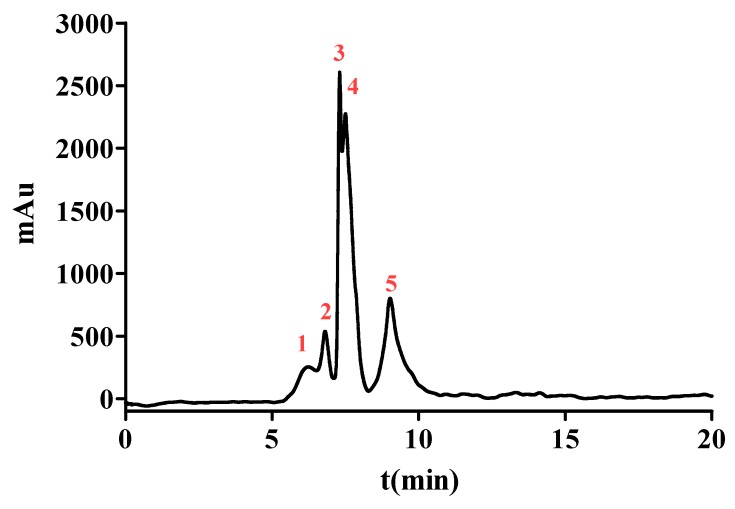
The chromatograms measured at 220 nm of SPD-VII by NGC Quest™ 10 Plus Chromatography System. The red numbers 1-5 represent peaks 1-5 respectively.

To enhance the ACE inhibition, SPDS-VII was further purified by NGC Quest™ 10 Plus Chromatography System. It is an effective way to purify proteins or peptides and collect them in the preparation phase. The chromatogram is shown in Figure 2. Five major peaks were eluted and collected for evaluating the ACE inhibition. As data represented in Table 2, peak 1 with the retention time from 5.11 to 6.52 min showed an IC_50_ value of (2.847 ± 0.045) μg/mL, peak 2 with the retention time from 6.52 to 7.12 min exhibited an IC_50_ value of (1.421 ± 0.035) μg/mL. The IC_50_ value of peak 3 with a retention time from 7.12 to 7.42 min was (1.838 ± 0.026) μg/mL, peak 4 with a retention time from 7.49 to 8.27 min was (0.558 ± 0.003) μg/mL and peak 5 with a retention time from 8.27 to 10.80 min was (0.757 ± 0.014) μg/mL. These results demonstrated that peak 4 possessed the most potent ACE inhibitory activity among the five peaks and thus was selected for further identification.

### 2.4. Characterization of ACE Inhibitory Peptide by Nano UHPLC-ESI-MS/MS from Peak 4

The peptides from peak 4 were identified by Nano UHPLC-ESI-MS/MS. The amino acid sequences from *Sesamum indicum* L. were found in Uniprot database (http://www.uniprot.org/). In this study, a total of 11 peptides were identified by analysis of the MS/MS spectrum (see Figure S1a–k). The sequences of the peptides were GHIITVAR, IGGIGTVPVGR, HIGNILSL, FMPGVPGPIQR, PNYHPSPR, AFPAGAAHW, HIITLGR, LAGNPAGR, MPGVPGPIQR, AGALGDSVTVTR, and INTLSGR, respectively. These peptides were chemically synthesized, and then their IC_50_ values inhibiting ACE activity were measured and the corresponding protein names are listed in Table 3. These peptides contained 7–12 amino acids with the molecular weight ranging from 754.8 to 1198.4 kDa; nevertheless, they had stronger ACE inhibitory activity in this study than the identified ACE inhibitory peptide YAHYSYA with the IC_50_ value of 0.6 mg/mL (686.58 μM) from sesame protein hydrolyzed by alcalase [20], suggesting that stronger ACE inhibitory activity was due to the action of endogenous enzymes in the body when simulating gastrointestinal digestion. Alternatively, sesame protein was hydrolyzed more thoroughly by multiple enzymes resulting in more cleavage sites via simulated gastrointestinal digestion in vitro. 

According to previous literature reports, most of the proteins present in sesame seeds are storage proteins composed of globulins (67.3%), albumins (8.6%), prolamines (1.4%), and glutelins (6.9%), of which sesame 11S globulins includes 11S globulin seed storage protein 2 precursor, 11S globulin isoform 3, and 11S globulin isoform 4 [28,29]. As shown in Table 3, there were five peptides derived from sesame 11S globulins, of which four were from11S globulin isoform 4 and one was from 11S globulin seed storage protein 2 precursor. This result indicated that sesame 11S globulins, especially 11S globulin isoform 4 mainly contributed to the generation of ACE inhibitory peptides. 

It is known that the ACE inhibition is strongly influenced by the type of amino acid composition of peptides. A series of studies have figured out that binding to ACE is influenced by hydrophobic amino acid residues (aromatic or branched chain) at three positions from the C-terminus of the peptide [30,31]. Some branched chain aliphatic amino acids, such as Ile and Val, are predominant in potent peptide inhibitors [32]. Moreover, the positively charged amino acids (Lys and Arg) have also been implied to increase the potency of ACE inhibitory peptides. Ryan et al. [33] concluded that hydrophilic peptides possess weak or no ACE inhibition. The hydrophobic nature of the N-terminus is another common feature of ACE inhibitory peptides. As a whole, the overall hydrophobicity of the peptide is important for ACE inhibitory activity. In the present study, a difference between MPGVPGPIQR (IC_50_ = 54.79 ± 0.37 μM) and FMPGVPGPIQR (IC_50_ = 11.08 ± 0.15 μM) in ACE inhibition further supported the above conclusion. Nevertheless, there were some exceptions, for example, the peptide EN and EVD from whey protein and the peptide CRQNTLGHNTQTSIAQ from *Stichopus horrens* do not have any reported specific amino acid at the C-terminal [34,35]. Thus, the relationship between the structure and function of ACE inhibitory peptides has not been fully established to date. 

There were nine peptides (GHIITVAR, IGGIGTVPVGR, FMPGVPGPIQR, PNYHPSPR, HIITLGR, LAGNPAGR, MPGVPGPIQR, AGALGDSVTVTR, INTLSGR) containing Arg in this study (Table 3). Arg is a basic aliphatic amino acid and also a positively charged amino acid, which was most frequently observed at the C-terminal of the ACE inhibitory peptides according to a lot of studies, such as YR and IR from the hydrolysates of marine sponge [36], APER from trevally hydrolysate [37]. It was noteworthy that the peptide GHIITVAR exhibiting the lowest IC_50_ value of 3.60 ± 0.10 μM among the 11 peptides. The inhibition potential of GHIITVAR was still less than the synthetic ACE inhibitor Captopril (IC_50_ = 0.0071 μM). GHIITVAR contained a hydrophobic amino acid Arg at the C-terminus and two aliphatic amino acids (Val and Ala) at the antepenultimate and penultimate positions, respectively. Besides, the presence of two aliphatic amino acids Ile may contribute to ACE inhibitory activity. However, the IC_50_ value of the peptide HIITLGR was 74.65 ± 0.13 μM, which further confirmed the fact that the presence of hydrophobic amino acids, especially those with aliphatic chains such as Gly at the N-terminus might enhance the ACE inhibition. 

In addition, the cleavage sites of pepsin were hydrophobic amino acids such as Phe, Trp, Leu, and Tyr. Trypsin preferentially cleaved at Arg and Lys and α-chymotrypsin preferentially cleaved at Trp, Tyr, and Phe. The cleavage site specificity of pepsin, trypsin, and α-chymotrypsin was found in bioinformatics tools (https://web.expasy.org/peptide_cutter/). From these results mentioned above, it could be concluded that the addition of trypsin and α-chymotrypsin significantly increase the exposure of active sites and thus increase ACE inhibitory activity. This was also consistent with the results in Figure 1c and explained why the inhibitory activity rose after pepsin digestion.

### 2.5. Molecular Docking Simulation between Peptide and ACE

**Figure 3:**
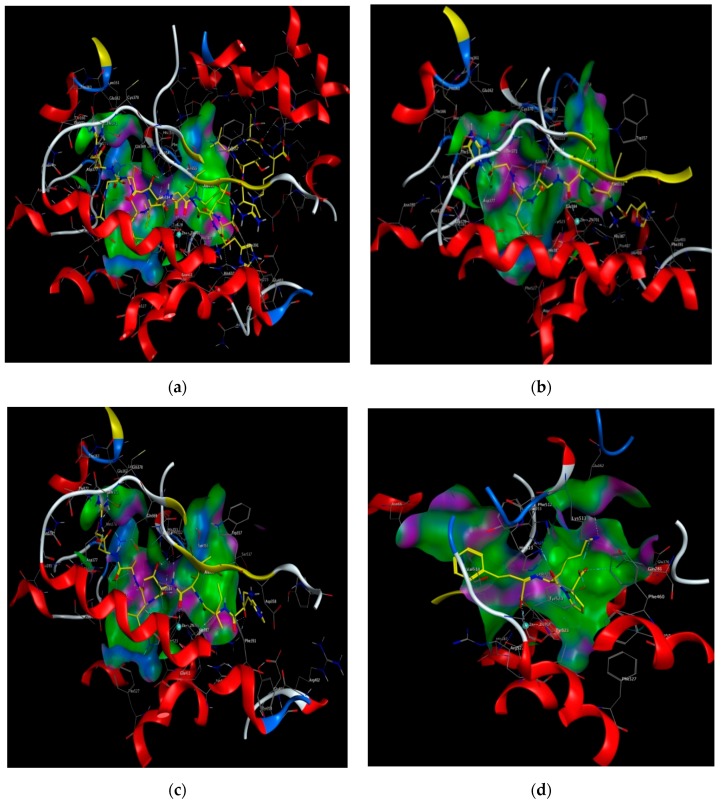
Computational visualization of the optimal docking conformation of AGALGDSVTVTR (**a**), HIITLGR (**b**), GHIITVAR (**c**), and lisinopril (**d**) with the active site of ACE. Carbon is in yellow, hydrogen is in grey, nitrogen is in dark blue, and oxygen is in red. The dotted lines represent hydrogen bonds.

Molecular docking was performed to estimate the binding affinities between peptide and ACE by using Surflex-Dock in Sybyl. In this study, lisinopril (5362119.pdb) was docked again with the prepared ACE. Taking GHIITVAR as an example, the C scores of the 20 conformations were different, but the Total scores gradually decreased (Figure S2). Therefore, the conformations of ranked No. 1 based on Total scores for each ligand were selected as the best docking conformation and the docking scores of 11 peptides and lisinopril were calculated. C score, as a synthetic scoring function was used for screening the binding affinity of ligand bound to ACE. As shown in Table 4, the Total score and C score of lisinopril were 11.24 and 4, respectively, which was in accordance with the results of the report basically [38]. Three peptides (AGALGDSVTVTR, HIITLGR, GHIITVAR) had the same highest C scores as lisinopril, whereas the C scores of the other peptides were below 4 (Table S1), suggesting that these peptides had stronger binding affinity with ACE. The computational visualization of the best docking conformation of the screened peptides and lisinopril by using Molecular Operating Environments (MOE; 2015.10 Chemical Computing Groups, Montreal, Quebec, Canada) is shown in Figure 3. Results revealed that these peptides were inserted into the binding site of ACE and formed hydrogen bonds with ACE residues. 

**Figure 4:**
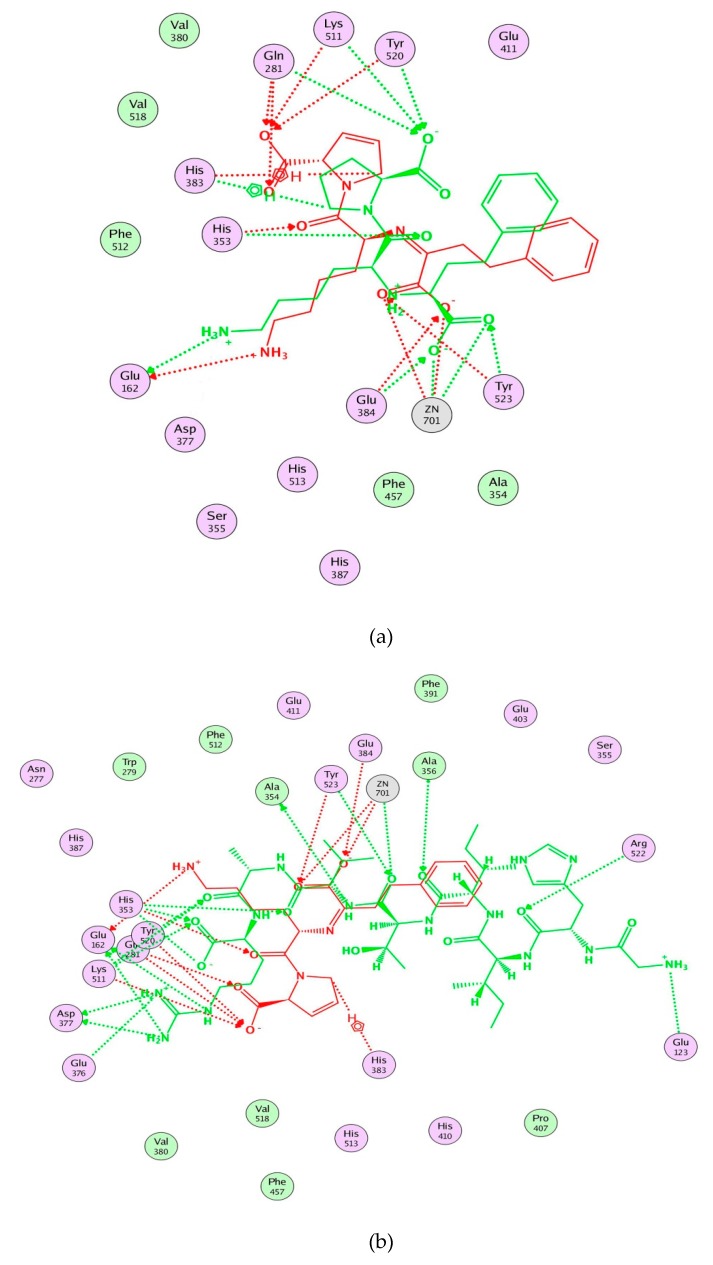

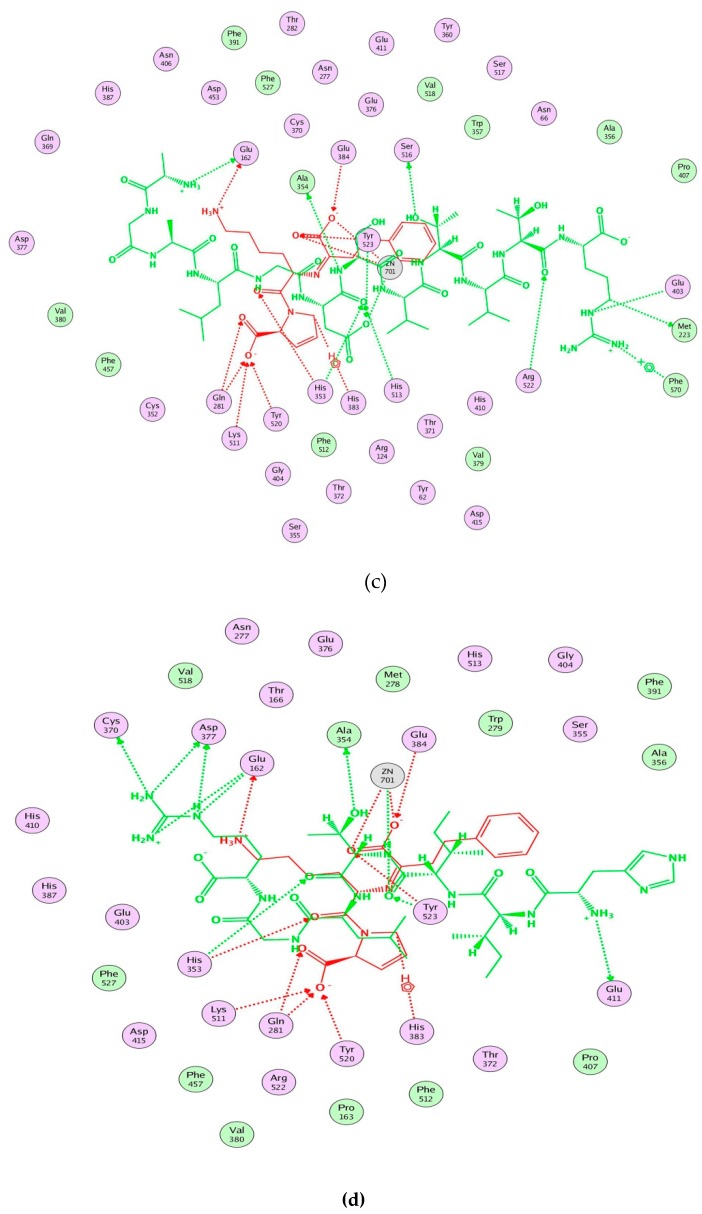
Molecular modeling of the interaction between inhibitors and ACE. (**a**) Molecular modeling of the interaction between lisinopril and ACE. (**b**) Molecular modeling of the interaction between GHIITVAR and ACE. (**c**) Molecular modeling of the interaction between AGALGDSVTVTR and ACE. (**d**) Molecular modeling of the interaction between HIITLGR and ACE. The ACE-lisinopril complex (1O86.pdb) was used as a template. The structure of lisinopril is in red and the peptides is in green. Hydrophobic, polar, and acidic residues of ACE are represented by green, violet, and red rings, respectively. Green and red arrows show hydrogen bonds from donor atom to acceptor.

Moreover, molecular modeling of the interaction between the peptides and ACE was further analyzed to explain the difference in the ACE inhibitory activities by using MOE. Figure 4 demonstrated that the results of the peptides’ interaction with ACE and lisinopril was taken as a reference. According to previous reports, the active sites of ACE were divided three main pockets (S1, S2, and S1′). S1 pocket includes Ala354, Glu384, and Tyr523 residues and S2 pocket includes Gln281, His353, Lys511, His513, and Tyr520 residues, while S1′contains Glu162 residue. ACE had a zinc ion (Zn^2+^) in its active site that coordinates with His383, His387, and Glu411 [39,40,41]. The hydrogen bonds play an important role in binding of the inhibitors to ACE potentially [41,42]. As shown in Figure 4a, lisinopril (5362119.pdb) was docked again with ACE and the interaction mode was the same as ACE-lisinopril complex (1O86.pdb), which indicated that the molecular docking procedure by using MOE was feasible. Lisinopril, as a synthetic antihypertensive drug, shared hydrogen bond interactions at Tyr523, Glu384, His353, Tyr520, Gln281, Lys511, Glu162, His383 and could bind the enzymatic active site through a metal ion interaction (Zn701). The peptide GHIITVAR displayed an excellent match with the active sites of ACE and formed hydrogen bonds with the S1 pocket (Ala354 and Tyr523), the S2 pocket (Gln281, His353, Tyr520, Lys511), and the S1′ pocket (Glu162) after docking. It also bound to the active sites using a metal ion interaction (Zn701) and a hydrogen bond interaction with His383. In addition, the six residues surrounding the ACE active site, Ala356, Arg522, Glu123, Asp377, and Glu376, contributed significantly in stabilization of this peptide-ACE complex. Therefore, it was not hard to see that GHIITVAR may have a competitive inhibition through binding at active sites when compared to lisinopril. As shown in Figure 4c, the peptide AGALGDSVTVTR was only able to interact with Ala354, His353, Glu162, Glu411, and coordinate with Zn^2+^. Similarly, the peptide HIITLGR displayed in Figure 4d established hydrogen bonds with Ala354, His353, Glu411, and coordinated with Zn^2+^. These results revealed that GHIITVAR could effectively interact with the active sites of ACE and might explain why this peptide exhibited exceptional ACE inhibition.

## 3. Discussion

Typically, bioactive peptides are delivered via oral administration. Therefore, it is crucial to study their changes during gastrointestinal digestion. These peptides by enzymatic hydrolysis of exogenous proteases may be easily metabolized or further hydrolyzed in the digestive system, which may result in reduced or increased potency, or to a complete loss of activity [43]. In the present study, the digestion in vitro with pepsin, trypsin, and α-chymotrypsin enzymes was applied to simulate the gastrointestinal digestion procedure. After simulated gastrointestinal digestion, the action of endogenous digestive enzymes increased the ACE inhibitory activity of sesame protein as a result of the release of smaller peptide fragments and additive and synergistic biological effects [44]. Compared with in vivo digestion, simulated gastrointestinal digestion in vitro has the advantages of controllable conditions, simple operation, good reproducibility, and cost saving. This method has been widely used to study the digestion and formation of functional peptides of dietary proteins in recent years. 

ACE inhibitory tripeptide generally requires a hydrophobic, a positively charged, and an aromatic amino acid residue at the N-terminal, middle, and C-terminal, respectively. Indeed, exact length of the C-terminal region to efficiently inhibit ACE activity remains unknown. Long-chain peptides could not always be speculated from that of their C-terminal tripeptide residues, implying the importance of other positions such as the fourth and so on [45]. García-Mora found that C-terminal heptapeptide was crucial for their antioxidant and ACE inhibitory activities [46]. 

Additionally, searching against the BIOPEP database (http://www.uwm.edu.pl/biochemia/index.php/pl/biopep) revealed that it is the first time the peptides with potent ACE inhibitory activity extracted from *Sesamum indicum* L were obtained. Therefore, sesame protein would be an attractive raw material generating ACE inhibitory peptides for controlling hypertension. Of course, further studies on the in vivo effects and constructing a three-dimensional quantitative structure–activity relationship (3D-QSAR) model still needs further investigation. 

## 4. Materials and Methods

### 4.1. Materials and Reagents

White sesame (*Sesamum indicum* L.) seeds were purchased from China Oil and Foodstuffs Corporation (Beijing, China). Angiotensin Converting Enzyme (ACE) from rabbit lung, N-Hippuryl-His-Leu (HHL) hydrate power (≥98%, HPLC), trifluoroacetic acid (TFA), 2,4,6 trinitrobenzene sulfonic acid (TNBS), and O-phthaldialdehyde (OPA), were purchased from Sigma-Aldrich (St. Louis, MO, USA). Pepsin (3000-3500 NFU/g), trypsin (250.N.F.U/mg), and α-chymotrypsin (1200 u/mg) were obtained from Solarbio Science and Technology Co., Ltd. (Beijing, China). Nanofiltration and ultrafiltration membranes were purchased from Rising Sun Membrane Technology Co., Ltd. (Beijing, China). All other reagents used in this study were of analytical grade.

### 4.2. Extraction of Sesame Protein

The extraction of sesame protein was carried out pursuant to the method demonstrated by Saatchi et al. [47] with several modifications. Sesame seeds were ground in a crusher and were extracted with petroleum ether to remove the fat. The defatted sesame meal was dissolved in distilled water at a solid/liquid (S/L) ratio of 1 g/20 mL and the pH of the suspension was adjusted to 11.0 by using 5 mol/L NaOH. Then it was stirred in a blender for 1 h and centrifuged at 5000 r/min for 20 min at 4 °C. The supernatant was collected, and the residue was repeatedly extracted under the above conditions. Finally, the supernatant was combined and the protein in the supernatant was precipitated by adjusting the pH to 4.3 with 5 mol/L HCL. The precipitate was collected, freeze dried, and stored at −20 °C for further use.

### 4.3. Preparation of in Vitro Simulated Gastrointestinal Digestion of Sesame Protein

The simulated gastrointestinal digestion process was modified slightly according to the method used by Sangsawad et al. [48]. Briefly, a certain amount of sesame protein was added to distilled water and stirred evenly to form an 8% protein solution. The solution was heated in a water bath at 95 °C for 30 min to denature the protein completely after neutralizing the pH to 7.0. In the simulated gastric digestion phase, the suspension was digested by pepsin (0.4% of substrate, dry basis) at 37 °C for 4 h during which time the pH was adjusted to 2.0 with HCL. During the simulated intestinal digestion phase, trypsin (0.3% of substrate, dry basis) and α-chymotrypsin (0.1% of substrate, dry basis) was added and the reaction was carried out at 37 °C for 6 h, during which time NaOH was used to keep the pH of the digested solution at 7.6. Samples were collected every 30 min during digestion for determination. After 10 h, the digested solution was cooled to room temperature and centrifuged at 5000 r/min for 20 min. The supernatant was collected and nanofiltrated through a flat-membrane test cell C60F (Nitto Denko Co., Ltd., Osaka, Japan) to remove the salt [29]. Nanofiltration membrane NF1 (MWCO of 150 Da) was put into the test cell. The 100 mL of the supernatant and 100 mL distilled water was poured into the test cell and then the nanofiltration began at pressure of 3.0 MPa and stirring speed of 400 rpm. When the volume of permeate reached 100 mL, the first nanofiltration terminated. Nanofiltration was repeated thrice. The retentate was collected as the final SPDS and stored at 4 °C for further separation and purification. 

### 4.4. Separation of ACE Inhibitory Peptide from SPDS by Ultrafiltration

The ACE inhibitory peptides from SPDS were separated by using MWCO of 100, 50, 30, 10, 5, and 3 kDa ultrafiltration membranes in a stirred cell C60F. The 250 mL of SPDS was poured into the test cell and then the ultrafiltration began at room temperature. The pressure was 2.5 MPa and the stirring speed was 400 rpm. When the permeate reached 200 mL, the first step ultrafiltration ended. Then the permeate was treated for the next step ultrafiltration under the above conditions. Each retentate was collected separately and seven fractions were generated, named SPDS-I (molecular weight over 100 kDa), SPDS-II (molecular weight between 50 and 100 kDa), SPDS-III (molecular weight between 30 and 50 kDa), SPDS-IV (molecular weight between 10 and 30 kDa), SPDS-V (molecular weight between 5 and 10 kDa), SPDS-VI (molecular weight between 3 and 5 kDa), and SPDS-VII (molecular weight below 3 kDa). These fractions were evaporated and lyophilized for subsequent ACE inhibitory study.

### 4.5. Purification of ACE Inhibitory Peptide by NGC Quest™ 10 Plus Chromatography System

The screened fraction with the strongest ACE inhibitory activity was purified by NGC Quest™ 10 Plus Chromatography System. First, 0.5 mL of the sample was filtered through a 0.22 µm membrane and injected into the system (Bio-Rad Laboratories, Hercules, CA, USA) with a Shim-pack GIS C18 preparative column (20 cm × 250 mm I.D., 10 μm, Shimadzu, Kyoto, Japan). The experiment was executed by using mobile phase A (ultrapure water containing 0.1% (*v/v*) TFA) and mobile phase B (methanol containing 0.1% (*v/v*) TFA). The sample was separated by a linear gradient elution from 55% to 60% of mobile phase A at a flow rate of 7.5 mL/min. The absorbance was monitored at 220 nm with a UV detector. Each chromatographic peak was pooled by repeated injection with corresponding test tube respectively through the fraction collector and lyophilized to determine the ACE inhibitory activity. 

### 4.6. Identification of the Purified ACE Inhibitory Peptide by Nano UHPLC-ESI-MS/MS

The eluted peak composition with the strongest ACE inhibitory activity was firstly performed by Thermo Ultimate 3000 UHPLC (Thermo Fisher Scientific,). The sample was enriched and salt-removed in a trap column, and then was injected onto the C_18_ column (25 cm × 75 µm I.D., 3 µm). The mobile phase A was 2% acetonitrile, 0.1% formic acid, and the mobile phase B was 98% acetonitrile, 0.1% formic acid. The separation was carried out at a flow rate of 300 NL/min through the following gradient elution: 0–5 min, 5% mobile phase B; 5–45 min, mobile phase B linearly increased from 5% to 25%; 45–50 min, mobile phase B increased from 25% to 35%; 50–52 min, mobile phase B increased from 35% to 80%; 52–54 min, 80% mobile phase B; and 54–60 min, 5% mobile phase B. 

Then mass analysis was performed on a Thermo Q-Exactive HF tandem mass spectrometer (Thermo Fisher Scientific, San Jose, CA, USA) after nano ESI ionization. The ion source voltage was set to 1.6 kV. The full scan range of mass spectrometry was 350–1600 m/z and the resolution was set to 60,000. The initial m/z of the secondary mass spectrometry was fixed at 100 and the resolution was set to 15,000. The ion fragmentation mode of high energy collisional dissociation (HCD) was set and the fragment ions were detected in Orbitrap, which was an ultrahigh field mass analyzer that could provide higher resolution and faster scanning speed. Databases including UniProt protein database, genomic annotation-based protein database, such as NCBI were selected for protein identification. The Mascot v2.3.02 software ((Matrix Sciences, London, UK) was used to process the MS/MS data for analyzing the peptide sequences.

### 4.7. Peptide Match and Peptide Synthesis

The protein names of the peptides were obtained from the Protein Information Resource (PIR) database (https://research.bioinformatics.udel.edu/peptidematch/index.jsp). Peptide match service is one of the most popular tools on the PIR website that plays an important role in locating occurrences of a specific peptide [49]. Meanwhile, in order to certify the ACE inhibitory activity of the purified peptides identified by mass spectroscopy, the selected peptides were chemically synthesized by GL Biochem Ltd. (Shanghai, China). The purity of the synthesized peptides reached 95% and was also verified by HPLC coupled with ESI-MS. These peptides were then dissolved in distilled water and assessed for ACE inhibitory activity.

### 4.8. Analysis of Degree of Hydrolysis

The degree of hydrolysis (DH) was determined according to the method of Adler-Nissen [50] with some modifications. Briefly, each sample at various points in time of simulated gastrointestinal digestion was appropriately diluted (125 µL) and then were mixed with phosphate buffer (1 mL, pH 8.2) and 1 mL of 0.1% TNBS reagent. The mixture was incubated at 50 °C for 60 min in the dark. At last, 2 mL of 0.1 mol/L HCL was used for the termination reaction and the samples were left at room temperature for 30 min. The standard curve was made with L-leucine and the absorbance of the reaction mixture was measured at 340 nm with a UV-visible spectrophotometer UV-3600Plus (Shimadzu Corporation, Kyoto, Japan). The degree of hydrolysis was determined from the following equation:

DH (%) = h/h_tot_ × 100 (1)

where h means concentration of peptide bond hydrolyzed (mmol/g) and h_tot_ means total amount of the peptide bond (8 mmol/g) [51].

### 4.9. Determination of Protein and Peptide Content 

The protein content before simulated gastrointestinal digestion was analyzed by Kjeldahl method as described by Marcó et al. [52]. The peptide content of each sample at various points in time of simulated gastrointestinal digestion was analyzed by using OPA spectrophotometric assay described by Agrawal et al. [53]. First, 40 mg of OPA was dissolved in 1 mL of methanol and mixed with 25 mL of 100 mmol/L sodium tetrahydroborate, 2.5 mL of 20% (*w/w*) sodium dodecyl sulfate, and 100 μL of β-mercaptoethanol. Distilled water was added to bring the total volume of the mixed solution up to 50 mL. Then the freshly prepared OPA solution was obtained. Subsequently, 100 μL of peptide solution was mixed with 2 mL of OPA solution. After the incubation for 2 min at room temperature, the absorbance of the reaction mixture was recorded spectrophotometrically at 340 nm by using a UV-visible spectrophotometer UV-3600Plus. Casein tryptone was used as a standard for quantification of peptide content. The peptide yield was determined by the ratio of peptide mass to protein mass.

### 4.10. Assay of the ACE Inhibitory Activity

The ACE inhibitory activity was measured by using cary eclipse fluorescence spectrophotometer (Agilent Technologies Australia Pty Ltd., Mulgrave, Australia) according to a modified method of Li et al. [54]. The sample was dissolved by distilled water to the appropriate concentration and the 96-well plate was used as the reaction vessel. Briefly, 30 μL of HHL substrate containing 0.3 mol/L NaCl in 50 mM borate buffer (pH 8.3) and 15 μL of samples were mixed. Then the reaction was started by adding 30 μL of ACE (12.5 mU/mL) to the mixture at 37 °C for 30 min. The reaction was stopped by using 125 μL of 1.2 mol/L NaOH. Finally, 30 μL of methanol solution containing 2% OPA was added, mixed evenly, and then stood at room temperature for 20 min. The derivative reaction was terminated by adding 40 μL of 6 mol/L HCl. The reaction solution was diluted 10 times and added into the fluorescence cell. The fluorescence absorption intensity was measured within 30–90 min. The determination conditions were as follows: the excitation wavelength was 340 nm, the emission wavelength was 455 nm, and the slit was 5 nm wide. Furthermore, the half inhibitory concentration (IC_50_) values, which meant the amount of inhibitor required to inactivate 50% of ACE activity under the experimental conditions were calculated from regression lines of a plot of % inhibition versus concentrations by using SPSS 13.0 software. All assays were performed in triplicate. ACE inhibitory activity was calculated by using the following equation:

ACE inhibitory activity (%) = [1 − (a−c)/(b−d)] × 100(2)

where a is the fluorescence absorption intensity in the presence of both ACE and inhibitor, b is the fluorescence absorption intensity in the presence of ACE but not inhibitor, c is the fluorescence absorption intensity in the presence of inhibitor but not ACE, d is the fluorescence absorption intensity in the absence of both ACE and inhibitor. 

### 4.11. Molecular Docking Simulation between Peptide and ACE

The molecular docking was carried out by using Surflex-Dock module that had an empirical scoring function and a patented searching engine [55,56] in Sybyl-X 2.1.1 software (Tripos, Inc., St. Louis, MO, USA). The 3D structure of the selected peptide was constructed, of which the atomic charge was calculated in MMFF94. Furthermore, the structure was energy minimized by using the Powell conjugate gradient optimization algorithm with a convergence criterion of 0.0005 kcal/mol Å and the maximum iterations of 10,000. The crystal structure of human ACE-lisinopril complex (1O86.pdb) was derived from the Protein Data Bank (http://www.rcsb.org/pdb/home/home.do). To prepare the structure of ACE, all substrates, chloride ions, and water molecules were excluded in ACE model. Then the polar hydrogens were added to the ACE model. The number of poses per ligand was set to 20 to perform the molecular docking and the conformations for each peptide based on Total scores of Surflex-Dock were ranked [38]. The conformations of ranked No. 1 for each peptide were selected and the other four kinds of docking score, D_score, G_score, PMF_score, and Chem score were estimated together using the C score module [57]. The interaction model between the ACE residues and peptides was analyzed by Molecular Operating Environments (MOE; 2015.10 Chemical Computing Groups, Montreal, Quebec, Canada). The ACE–lisinopril complex (1O86.pdb) was used as a template. In the docking process, semiflexible docking mode was adopted to treat the receptor and ligand, which meant that the conformation of receptor was rigid and that of ligand was flexible. 

### 4.12. Statistical Analysis

All analyses were performed in triplicate. Data was expressed as the mean ± standard deviation and performed by one-way analysis of variation by using SPSS 13.0 for Windows (Chicago, IL, USA) with Least Significant Difference (LSD) multiple range tests. The significant difference level was set at *p* < 0.05. 

## 5. Conclusions

In conclusion, this study isolated and purified new ACE inhibitory peptides from sesame protein by using two stages of simulated gastrointestinal digestion in vitro. The DH, peptide yield, and ACE inhibition at different time points were determined during simulated gastrointestinal digestion, which demonstrated that the DH, peptide yield increased with the increase of digest time. The action of endogenous digestive enzymes increased the ACE inhibitory activity after simulated gastrointestinal digestion. SPDS was purified by ultrafiltration and SPDS-VII (<3 kDa) had strongest ACE inhibition. SPDS-VII was further purified by NGC Quest™ 10 Plus Chromatography System and finally 11 major peptides were identified by Nano UHPLC-ESI-MS/MS from peak 4. The peptide GHIITVAR derived from 11S globulin exhibited superior ACE inhibitory activity with the IC_50_ value of (3.60 ± 0.10) μM among the peptides. Molecular docking results suggested that the ACE inhibition of GHIITVAR was mainly attributed to forming very strong hydrogen bonds with the S1 pocket (Ala354 and Tyr523), the S2 pocket (Gln281, His353, Tyr520, Lys511) and the S1’ pocket (Glu162) after docking. It also bound to the active sites using a metal ion interaction (Zn701) and a hydrogen bond interaction with His383. Based on these results, sesame protein can be a promising ACE inhibitor in the prevention and treatment of hypertension. In addition, GHIITVAR can be used as a potential nutraceutical for development of functional foods. 

## Figures and Tables

**Table 1:** Results of IC_50_ values of fractions from sesame protein digestive solution (SPDS) on ACE inhibitory activity.

Fraction	IC_50_ Values (μg/mL)
SPDS-I (>100 kDa)	35.143 ± 1.122 ^a^
SPDS-II (50–100 kDa)	15.066 ± 0.042 ^b^
SPDS-III (30–50 kDa)	9.146 ± 0.005 ^c^
SPDS-IV (10–30 kDa)	6.108 ± 0.001 ^cd^
SPDS-V (5–10 kDa)	5.106 ± 0.003 ^ce^
SPDS-VI (3–5 kDa)	4.583 ± 0.003 ^cf^
SPDS-VII (<3 kDa)	2.720 ± 0.003 ^cg^

Values are expressed as the mean ± standard deviation (*n* = 3). Values with different letters are significantly different (*p* < 0.05).

**Table 2:** The IC_50_ values inhibiting ACE activity of peaks 1-5 eluted from SPDS-VII.

Fraction	Retention Time (min)	IC_50_ Values (μg/mL)
peak 1	5.11–6.52	2.847 ± 0.045 ^a^
peak 2	6.52–7.12	1.421 ± 0.035 ^c^
peak 3	7.12–7.42	1.838 ± 0.026 ^b^
peak 4	7.49–8.27	0.558 ± 0.003 ^e^
peak 5	8.27–10.80	0.757 ± 0.014 ^d^

Values are expressed as the mean ± standard deviation (*n* = 3). Values with different letters are significantly different (*p* < 0.05).

**Table 3:** Peptides identified from peak 4 and corresponding physicochemical characteristics.

Peptide	Protein Name	Molecular Weight	IC_50_ Value (μM)
GHIITVAR	11S globulin isoform 4 (Q2XSW6_SESIN)	866.0	3.60 ± 0.10 ^k^
IGGIGTVPVGR	Elongation factor 1-alpha-like	1025.2	6.97 ± 0.18 ^j^
HIGNILSL	TBCC domain-containing protein 1	866.0	36.69 ± 0.33 ^f^
FMPGVPGPIQR	Oil body-associated protein 1A	1198.4	11.08 ± 0.15 ^i^
PNYHPSPR	11S globulin seed storage protein 2 precursor (Q9XHP0_SESIN)	967.0	18.98 ± 0.26 ^h^
AFPAGAAHW	11S globulin isoform 4 (Q2XSW6_SESIN)	927.0	29.00 ± 0.20 ^g^
HIITLGR	Protein NDH-DEPENDENT CYCLIC ELECTRON FLOW 5	808.9	74.65 ± 0.13 ^d^
LAGNPAGR	11S globulin isoform 4 (Q2XSW6_SESIN)	754.8	148.41 ± 0.35 ^b^
MPGVPGPIQR	Oil body-associated protein 1A	1051.2	54.79 ± 0.37 ^e^
AGALGDSVTVTR	60S ribosomal protein L22-2-like	1146.2	68.49 ± 0.14 ^c^
INTLSGR	11S globulin isoform 4 (Q2XSW6_SESIN)	759.8	149.63 ± 0.33 ^a^

Values are expressed as the mean ± standard deviation (*n* = 3). Values with different letters are significantly different (*p* < 0.05).

**Table 4:** Surflex-Dock scores (kcal/mol) of peptides and lisinopril.

Peptide	Total_Score ^1^	Crash ^2^	Polar ^3^	D_score ^4^	PMF_Score ^5^	G_Score ^6^	Chem Score ^7^	C Score ^8^
AGALGDSVTVTR	13.66	−7.05	11.00	−395.98	−306.63	−707.54	−41.93	4
HIITLGR	12.35	−6.48	8.63	−288.74	−264.56	−519.33	−26.18	4
GHIITVAR	10.03	−9.60	6.79	−357.58	−308.79	−685.80	−40.87	4
Lisinopril	11.24	−2.48	7.27	−160.13	−179.32	−284.82	−23.95	4

^1^ Total_score: represents the total surflex dock score expressed as ratio of concentrations of a compound in a mixture of two immiscible phases at equilibrium (−logKd). ^2^ Crash: stands for the capacity of penetration of a ligand into the active site of the protein. Crash scores close to 0 are favorable. Negative numbers indicate penetration. ^3^ Polar: describes the polar interaction of protein and the ligand. ^4^ D_score: stands for van der waals interaction between protein and the ligand. ^5^ PMF_score: (Potential of Mean Force, PMF) the free energies of interactions for protein-ligand atom pairs. ^6^ G_score: is based on hydrogen bonding, ligand-protein complex, and internal (ligand-ligand) energies. ^7^ Chem score: includes provisions for hydrogen bonding, lipophilic contact, and rotational entropy, metal-ligand interaction, along with an intercept term. ^8^ C score (the consensus score): gives a number of scoring functions of affinity of ligand bound to protein.

## Supplementary Materials

Supplementary materials can be found at https://www.mdpi.com/1422-0067/21/3/1059/s1.

## Abbreviations

ACE	Angiotensin I-converting enzyme
Nano UHPLC-ESI-MS/MS	Nano ultra-high performance liquid chromatography-electrospray ionization mass spectrometry/mass spectrometry
SPD	Sesame protein digestive solution
DH	Degree of hydrolysis
MWCO	Molecular weight cut-off
RAS	Renin-angiotensin system
KKS	Kallikrein kinin system
MOE	Molecular Operating Environments
