# Supplementary material for: Novel ACE Inhibitory Peptides Derived from Simulated Gastrointestinal Digestion in Vitro of Sesame (Sesamum indicum L.) Protein and Molecular Docking Study
Source: Int J Mol Sci. 2020 Feb 5;21(3):1059. doi: 10.3390/ijms21031059 (PMC7037947; doi:10.3390/ijms21031059)
Supplement: Supplementary file 1 [file ijms-21-01059-s001.pdf]

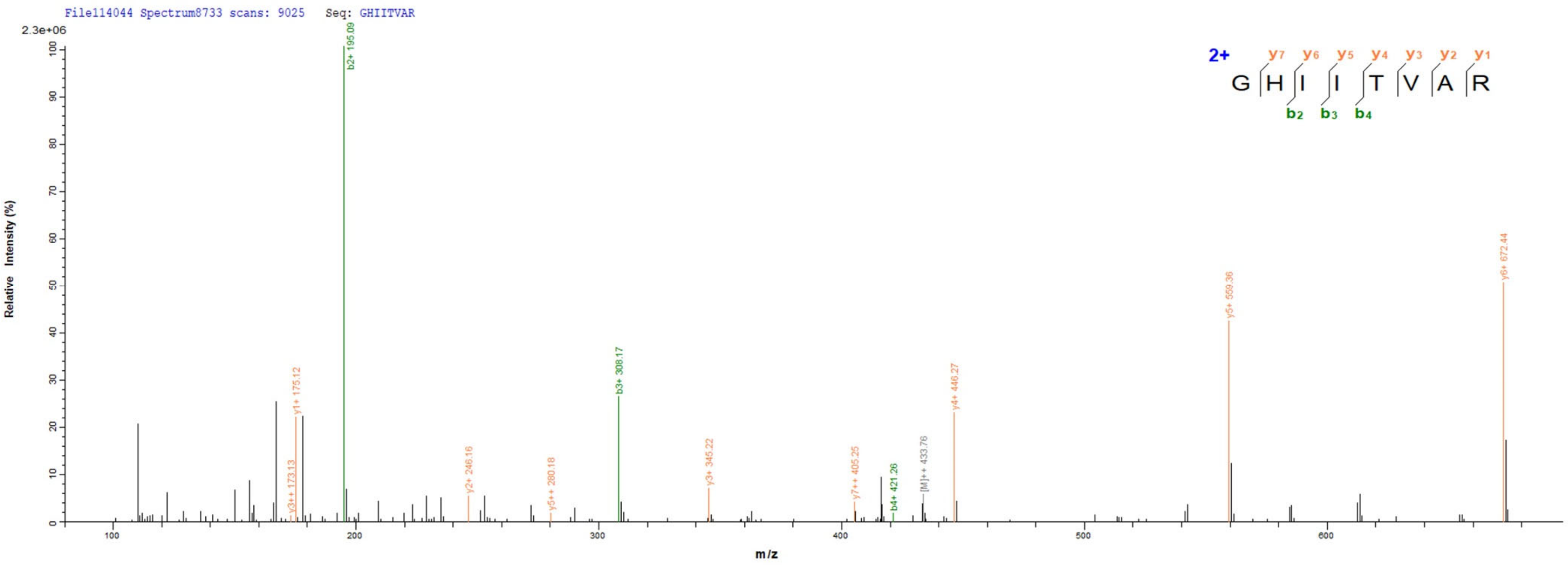
File114044 Spectrum8733 scans: 9025 Seq: GHIITVAR
2.3e+06
Relative Intensity (%)
m/z
2+
y7 y6 y5 y4 y3 y2 y1
G H I I T V A R
b2 b3 b4
b2+ 195.09
b3+ 308.17
y1+ 175.12
y3++ 173.13
y2+ 246.16
y5++ 280.18
y3+ 345.22
y7++ 405.25
b4+ 421.26
[M]++ 433.76
y4+ 446.27
y5+ 559.36
y6+ 672.44

(**a**)

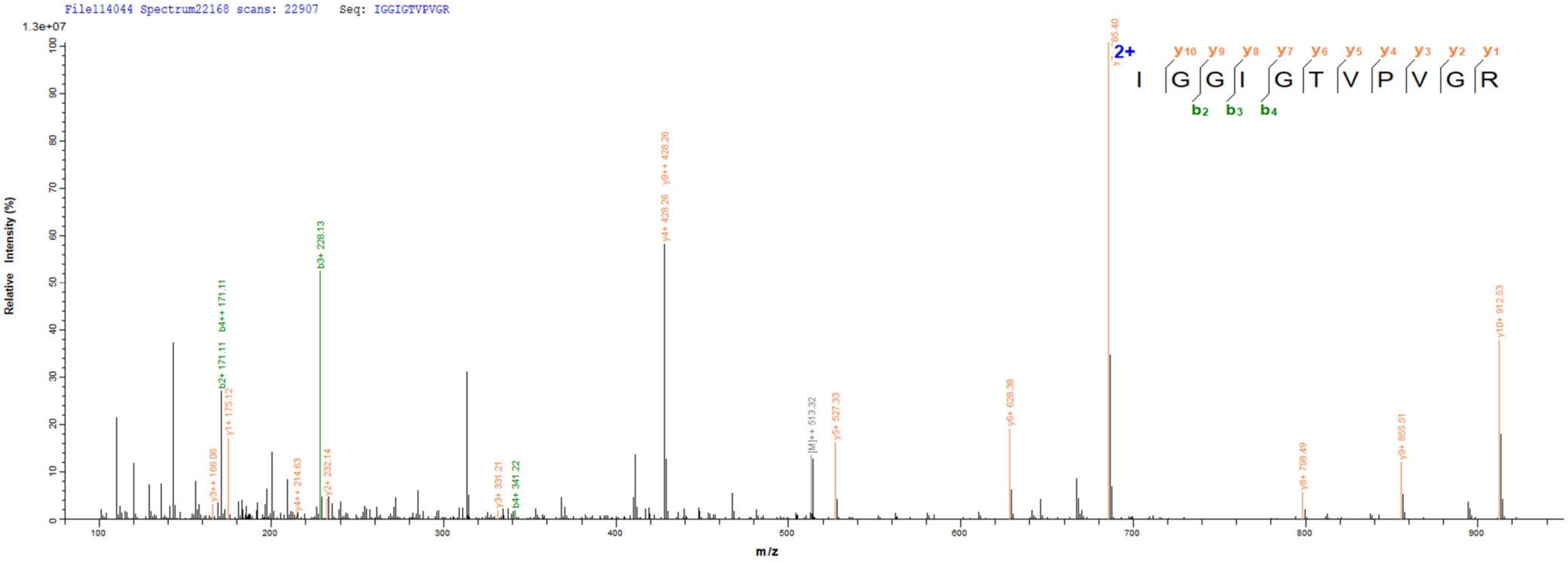
File114044 Spectrum22168 scans: 22907 Seq: IGGIGTVPVGR
1.3e+07
Relative Intensity (%)
m/z
2+
y10 y9 y8 y7 y6 y5 y4 y3 y2 y1
I G G I G T V P V G R
b2 b3 b4
y9++ 428.26
y4+ 428.26
b3+ 228.13
b4++ 171.11
b2+ 171.11
y1+ 175.12
y3++ 166.06
y4++ 214.63
y2+ 232.14
y3+ 331.21
b4+ 341.22
[M]++ 513.32
y5+ 527.33
y6+ 628.38
y8+ 798.49
y9+ 855.51
y10+ 912.53

(**b**)

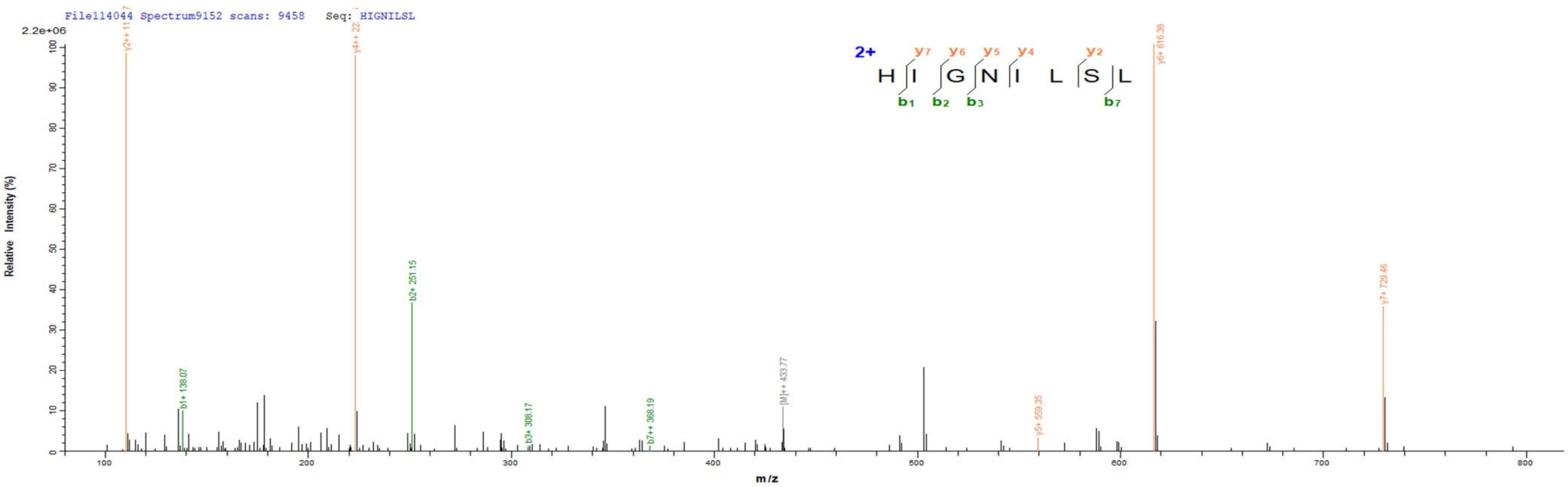
File114044 Spectrum9152 scans: 9458 Seq: HIGNILSL
2.2e+06
Relative Intensity (%)
m/z
2+
H I G N I L S L
y7 y6 y5 y4 y2
b1 b2 b3 b7
b1+ 138.07
b2+ 251.15
b3+ 308.17
b7++ 368.19
[M]++ 433.77
y5+ 559.35
y6+ 616.38
y7+ 729.46

(**c**)

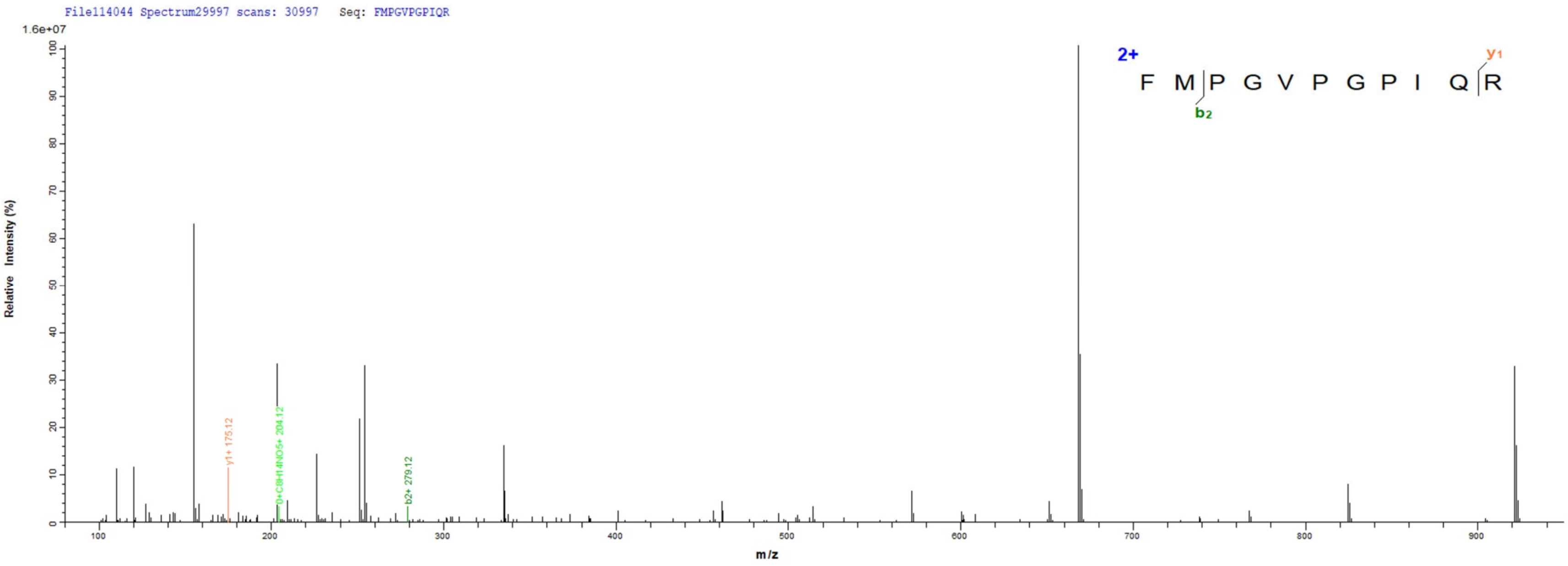
File114044 Spectrum29997 scans: 30997 Seq: FMPGVPGPIQR
1.6e+07
Relative Intensity (%)
y1+ 175.12
0+C8H14NO5+ 204.12
b2+ 279.12
2+
F M P G V P G P I Q R
b2
y1
m/z

(**d**)

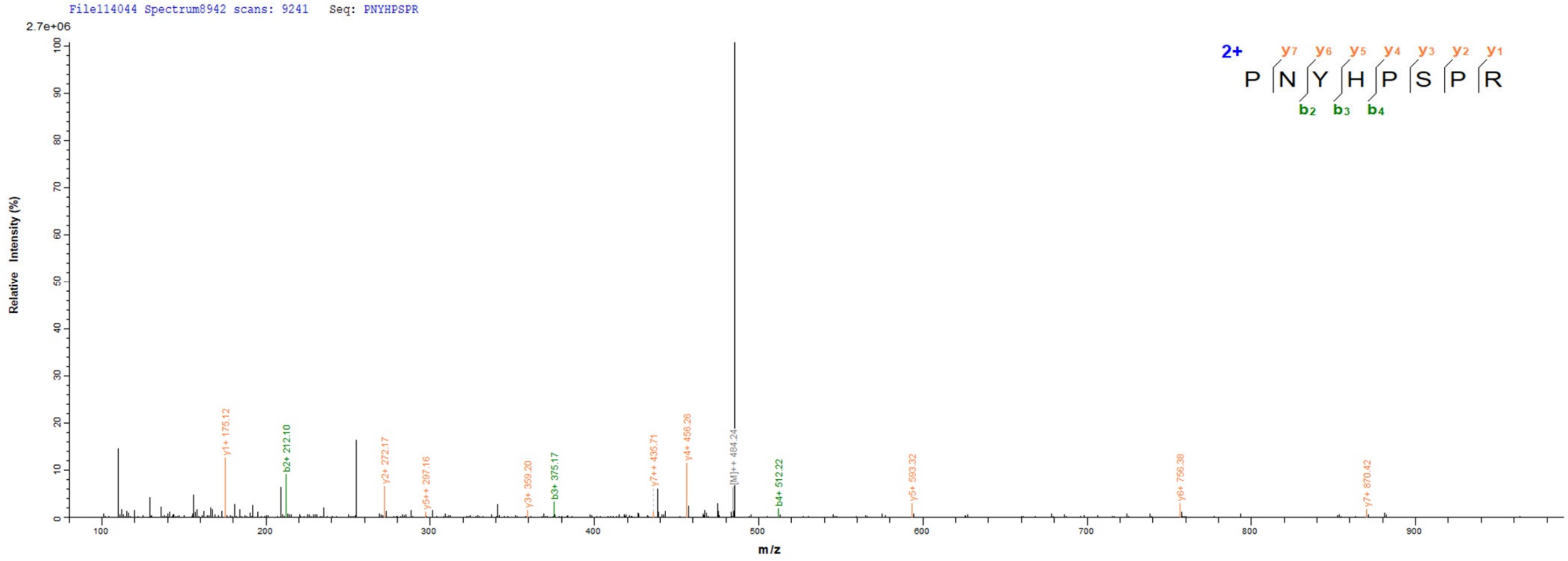
File114044 Spectrum8942 scans: 9241 Seq: PNYHPSPR
2.7e+06
Relative Intensity (%)
m/z
2+
P N Y H P S P R
y7 y6 y5 y4 y3 y2 y1
b2 b3 b4
y1+ 175.12
b2+ 212.10
y2+ 272.17
y5++ 297.16
y3+ 359.20
b3+ 375.17
y7++ 435.71
y4+ 456.26
[M]++ 484.24
b4+ 512.22
y5+ 593.32
y6+ 756.38
y7+ 870.42

(**e**)

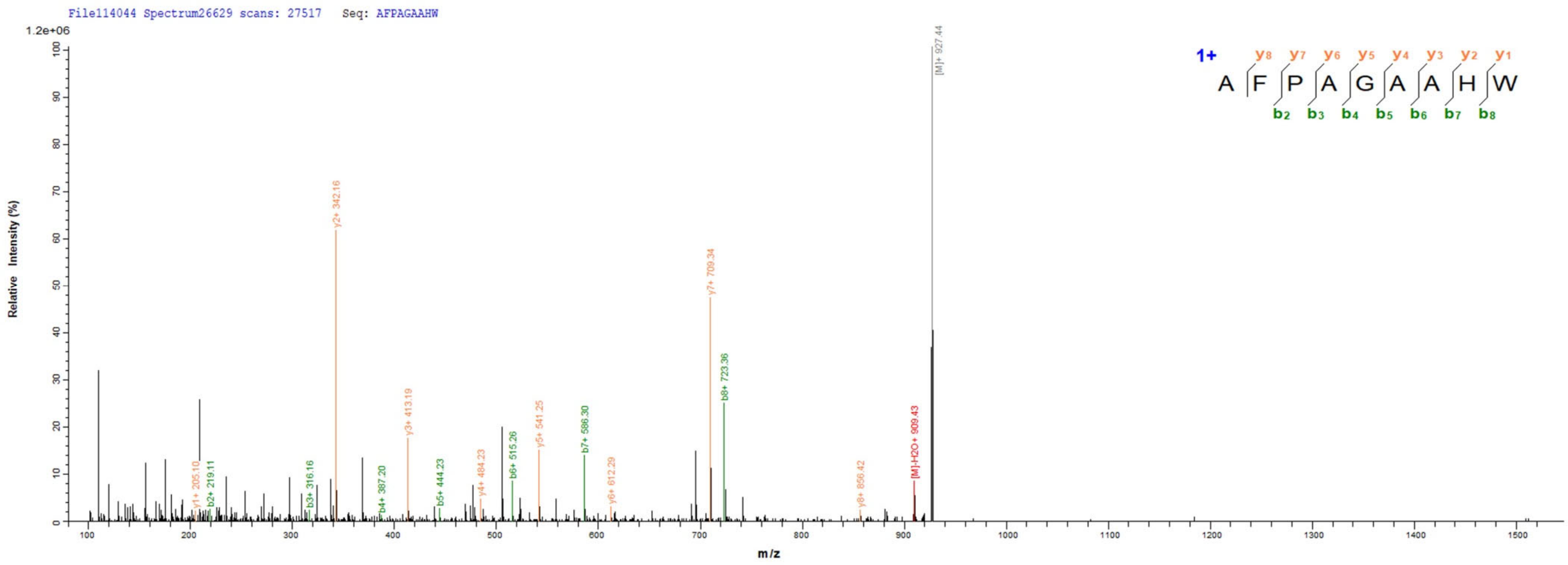

File114044 Spectrum26629 scans: 27517 Seq: AFPAGAAHW
1.2e+06
Relative Intensity (%)
m/z
1+
AFPAGAAHW
[M]+ 927.44
y2+ 342.16
y7+ 709.34
b8+ 723.36
y3+ 413.19
y5+ 541.25
b7+ 586.30
b6+ 515.26
y4+ 484.23
b5+ 444.23
b4+ 387.20
b3+ 316.16
b2+ 219.11
y1+ 205.10
y6+ 612.29
y8+ 856.42
[M]-H2O+ 909.43

(f)

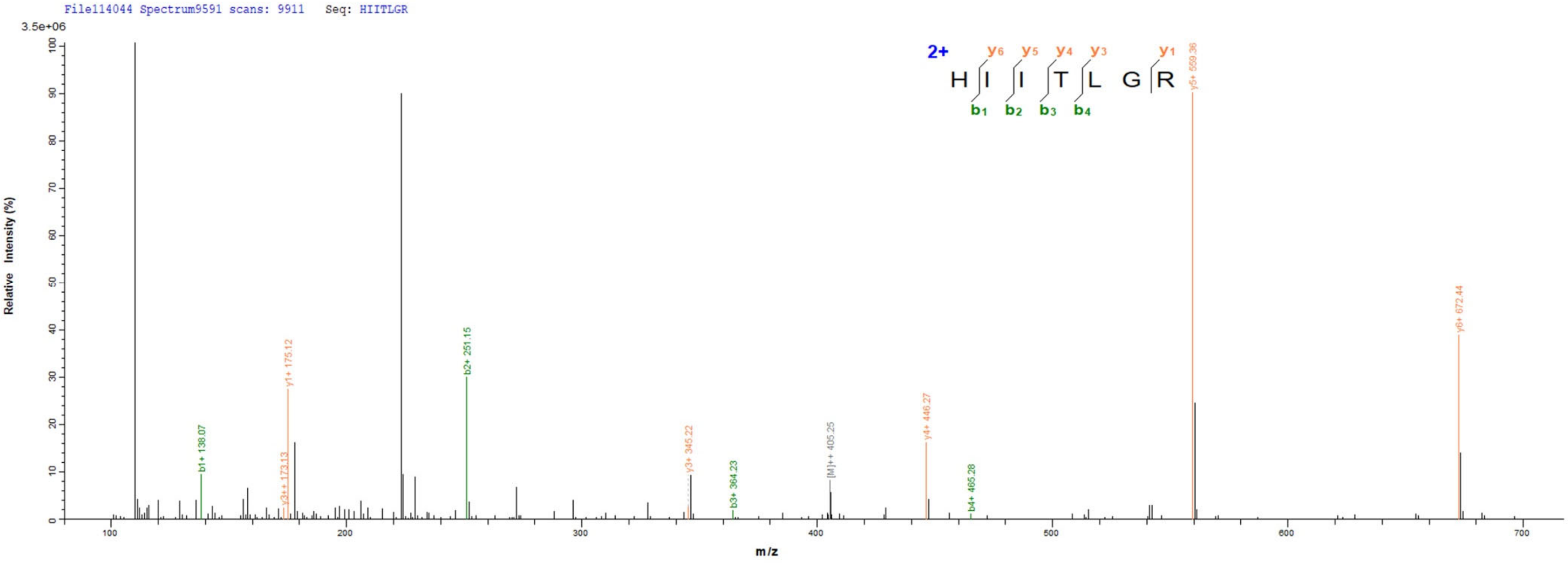
File114044 Spectrum9591 scans: 9911 Seq: HIITLGR
3.5e+06
Relative Intensity (%)
m/z
2+
H I I T L G R
y6 y5 y4 y3 y1
b1 b2 b3 b4
b1+ 138.07
y3++ 173.13
y1+ 175.12
b2+ 251.15
y3+ 345.22
b3+ 364.23
[M]++ 405.25
y4+ 446.27
b4+ 465.28
y5+ 559.36
y6+ 672.44

(g)

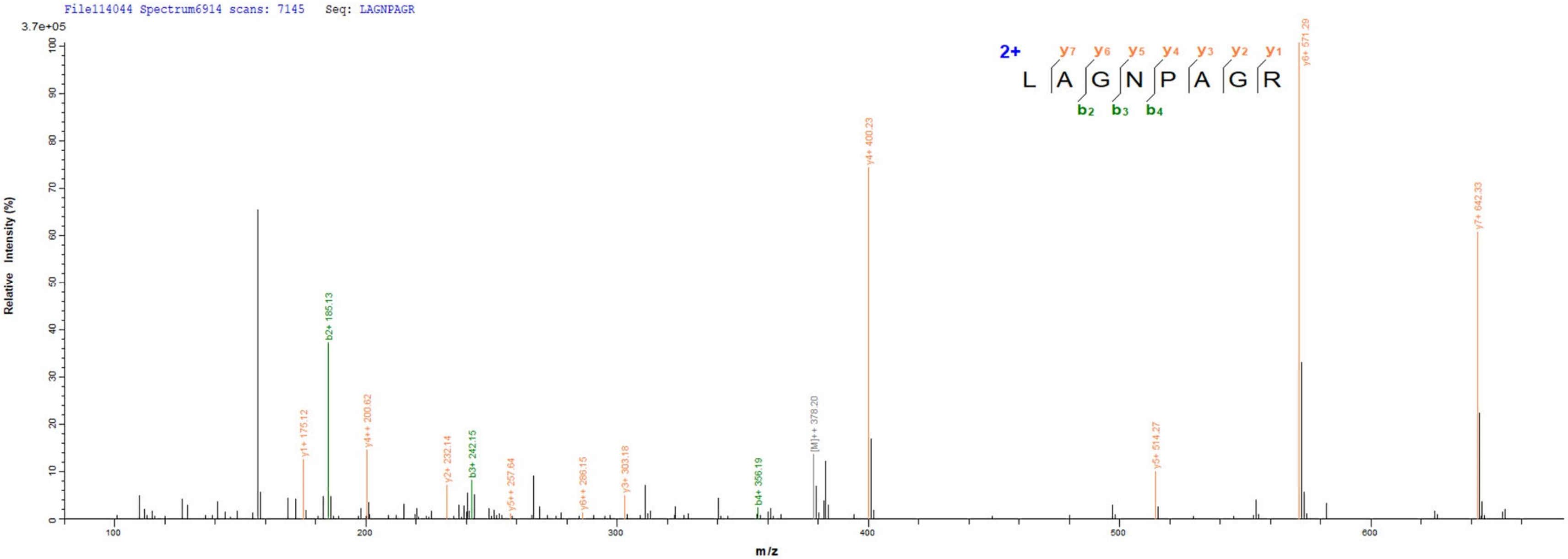
File114044 Spectrum6914 scans: 7145 Seq: LAGNPAGR
3.7e+05
Relative Intensity (%)
m/z
2+
LAGNPAGR
y1+ 175.12
b2+ 185.13
y4++ 200.62
y2+ 232.14
b3+ 242.15
y5++ 257.64
y6++ 286.15
y3+ 303.18
b4+ 356.19
[M]++ 378.20
y4+ 400.23
y5+ 514.27
y6+ 571.29
y7+ 642.33

(h)

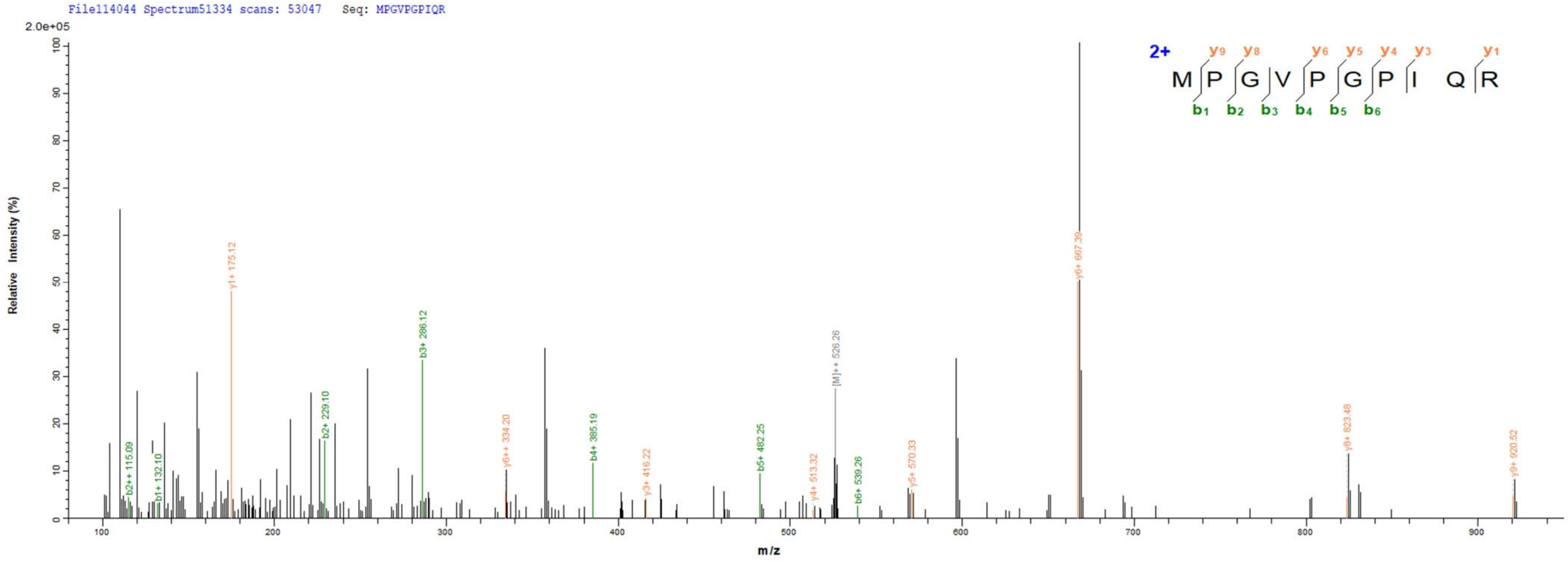

File114044 Spectrum51334 scans: 53047 Seq: MPGVPGPIQR
2.0e+05
Relative Intensity (%)
m/z
2+
MPGVPGPIQR
y1+ 175.12
b2++ 115.09
b1+ 132.10
b2+ 229.10
b3+ 286.12
y6++ 334.20
b4+ 385.19
y3+ 416.22
b5+ 482.25
y4+ 513.32
[M]++ 526.26
b6+ 539.26
y5+ 570.33
y6+ 667.39
y8+ 823.48
y9+ 920.52

(i)

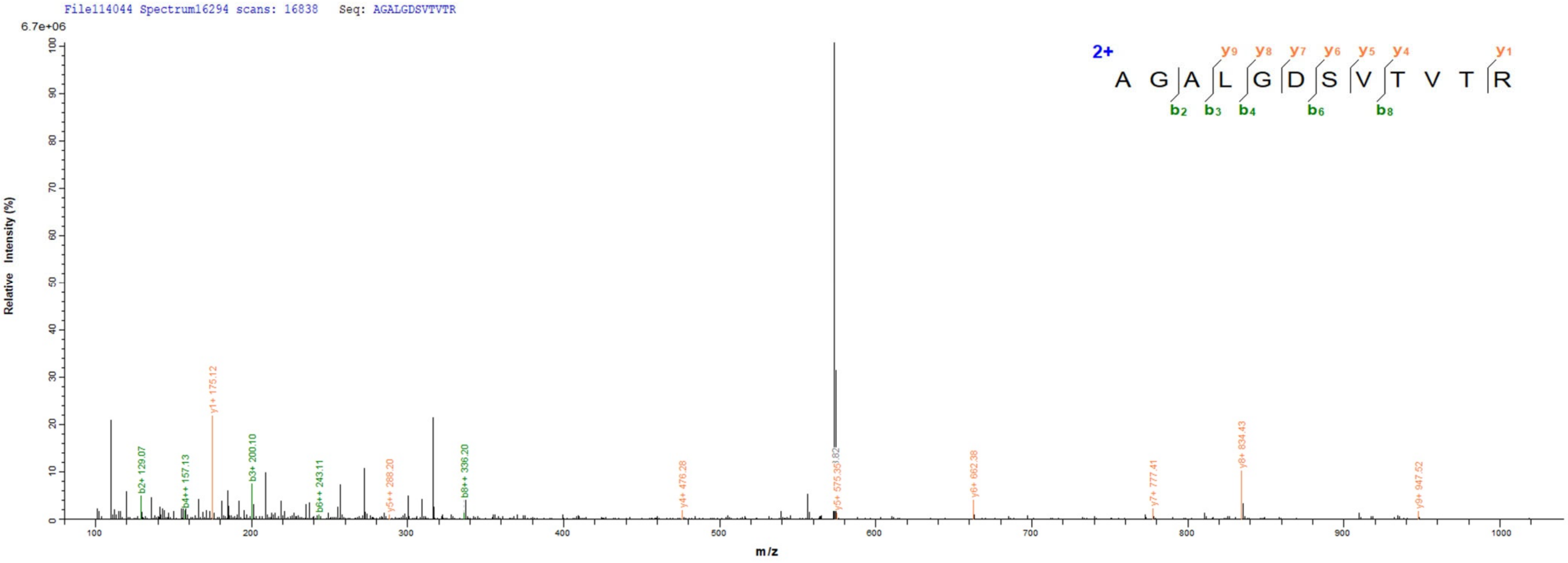
File114044 Spectrum16294 scans: 16838 Seq: AGALGDSVTVTR
6.7e+06
Relative Intensity (%)
m/z
2+
A G A L G D S V T V T R
y9 y8 y7 y6 y5 y4 y1
b2 b3 b4 b6 b8
y1+ 175.12
b2+ 129.07
b4++ 157.13
b3+ 200.10
b6++ 243.11
y5++ 288.20
b8++ 336.20
y4+ 476.28
y5+ 575.35
y6+ 662.38
y7+ 777.41
y8+ 834.43
y9+ 947.52

(j)

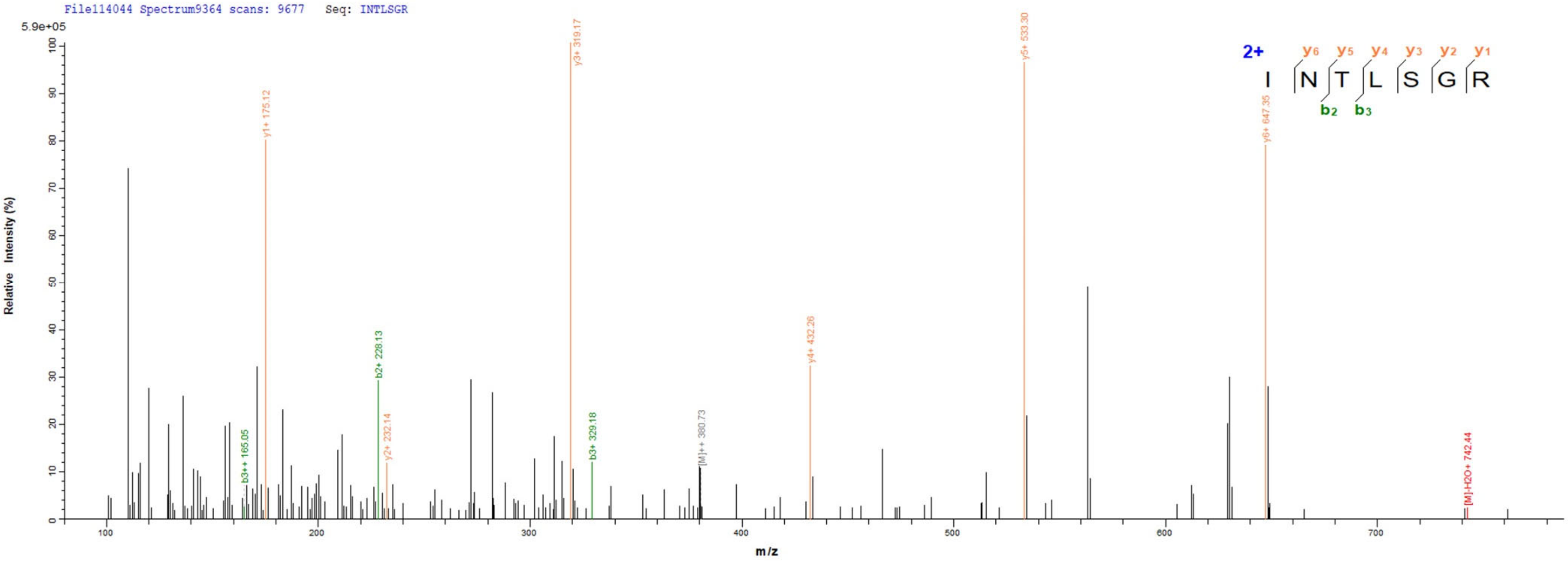

(k)

Figure S1. Identification of the molecular weight and amino acid sequence from peak 4. (**a**) MS/MS spectrum of GHIITVAR. (**b**) MS/MS spectrum of IGGIGTVPVGR. (**c**) MS/MS spectrum of HIGNILSL. (**d**) MS/MS spectrum of FMPGVPGPIQR. (**e**) MS/MS spectrum of PNYHPSPR. (**f**) MS/MS spectrum of AFPAGAAHW. (**g**) MS/MS spectrum of HIITLGR. (**h**) MS/MS spectrum of LAGNPAGR. (**i**) MS/MS spectrum of MPGVPGPIQR. (**j**) MS/MS spectrum of AGALGDSVTVTR. (**k**) MS/MS spectrum of INTLSGR.

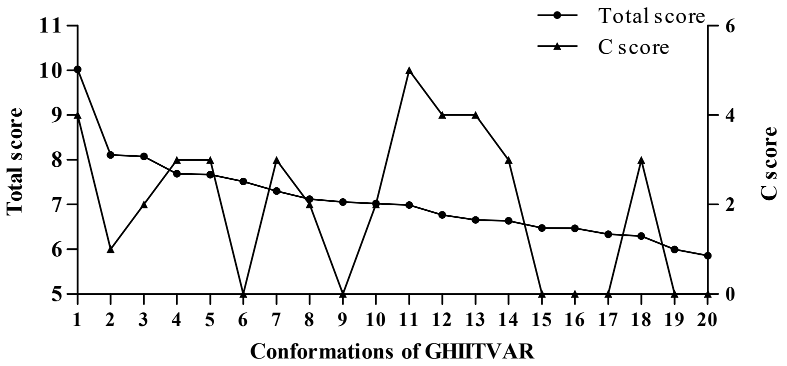

Figure S2. Total scores and C scores of comformations of GHIITVAR.

Table S1. Surflex-Dock scores (kcal/mol) of the other peptides.

Peptide	Total_score	Crash	Polar	D_score	PMF_score	G_score	Chem score	C score
INTLSGR	12.2544	−7.2199	12.0071	−317.9095	−275.737	−483.6159	−35.7317	1
LAGNPAGR	14.3901	−5.604	10.4595	−316.4394	−209.338	−527.4342	−42.3209	1
MPGVPGPIQR	12.5911	−5.6723	6.0252	−347.0212	−220.822	−649.0413	−10.4718	2
AFPAGAAHW	9.2034	−4.679	4.4847	−287.4604	−283.761	−507.8476	−41.4304	1
PNYHPSPR	10.0883	−6.7967	5.6331	−349.9793	−308.285	−674.6738	−35.9565	2
HIGNILSL	13.1644	−4.6353	9.2204	−307.7014	−236.307	−564.3041	−45.3885	0
FMPGVPGPIQR	9.6726	−7.723	7.2321	−371.4869	−263.845	−628.7991	−19.6976	3
IGGIGTVPVGR	11.964	−7.5337	8.7111	−363.7118	−269.274	−674.8707	−52.8792	1
